# Regulation and pharmacological targeting of RAD51 in cancer

**DOI:** 10.1093/narcan/zcaa024

**Published:** 2020-09-25

**Authors:** McKenzie K Grundy, Ronald J Buckanovich, Kara A Bernstein

**Affiliations:** Department of Pharmacology and Chemical Biology, University of Pittsburgh School of Medicine, Pittsburgh, PA 15213, USA; Division of Hematology Oncology, Department of Internal Medicine, University of Pittsburgh, Pittsburgh, PA 15213, USA; Division of Gynecologic Oncology, Department of Obstetrics and Gynecology, Hillman Cancer Center, University of Pittsburgh, Pittsburgh, PA 15213, USA; Department of Pharmacology and Chemical Biology, University of Pittsburgh School of Medicine, Pittsburgh, PA 15213, USA

## Abstract

Regulation of homologous recombination (HR) is central for cancer prevention. However, too little HR can increase cancer incidence, whereas too much HR can drive cancer resistance to therapy. Importantly, therapeutics targeting HR deficiency have demonstrated a profound efficacy in the clinic improving patient outcomes, particularly for breast and ovarian cancer. RAD51 is central to DNA damage repair in the HR pathway. As such, understanding the function and regulation of RAD51 is essential for cancer biology. This review will focus on the role of RAD51 in cancer and beyond and how modulation of its function can be exploited as a cancer therapeutic.

## INTRODUCTION

Unrepaired DNA damage can result in genome instability and cancer. Cancer prevention depends on the maintenance of several DNA damage repair pathways, including homologous recombination (HR). Tumors that are deficient in HR are sensitive to cancer therapeutics that interfere with DNA replication (1). In contrast, induction of DNA damage repair proteins is associated with therapeutic resistance and even metastasis (2). A key protein of the HR pathway is RAD51. RAD51 belongs to the *recA*/*RAD51* gene family that arose from a gene duplication of the archaeal RadA protein and is highly conserved throughout evolution (3–5). RAD51 is regulated by a group of proteins that include BRCA2, PALB2 and the RAD51 paralogs (6). Misregulation of RAD51, or one of its regulators, is associated with cancer as well as Fanconi anemia (FA)-like syndrome (6,7). While loss or reduction of RAD51 protein function can increase cancer risk, RAD51 upregulation in cancer can also contribute to therapeutic resistance (8,9). Maintaining appropriate levels of RAD51 expression and activity is critical for HR and thus cancer prevention.

## RAD51 AND DNA DOUBLE-STRAND BREAK REPAIR

**Figure 1.**
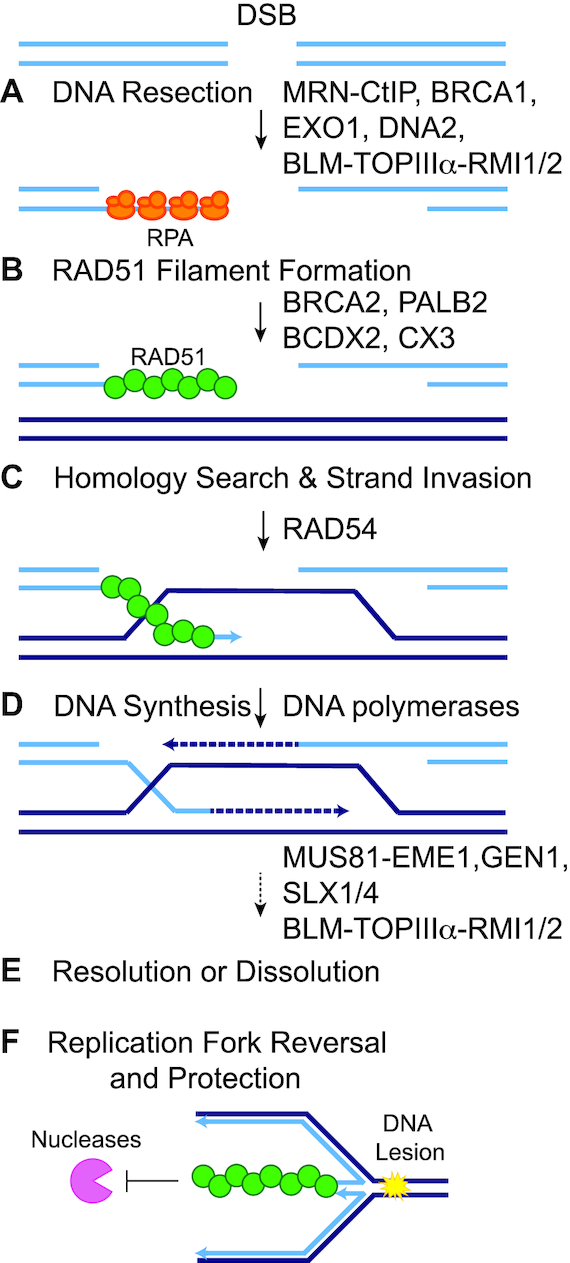
Schematic of RAD51 function during HR and replication fork reversal and protection. After a DSB occurs, the cell can use the HR pathway to repair the break using a homologous template (dark blue lines). (**A**) The DNA ends are resected to form 3′ ssDNA overhangs that are coated with RPA (orange ovals). Short-range DNA end resection is mediated by MRE11–RAD50–NBS1 (MRN) with CtIP and long-range DNA end resection is mediated by EXO1 or BLM–TOPIIIα–RMI1/2 with DNA2. BRCA1 also has an important function during DNA end resection. (**B**) RPA is then displaced by RAD51 (green circles), which subsequently forms a nucleoprotein filament. RAD51 filament formation is aided by PALB2, BRCA2 and the RAD51 paralog sub-complexes (BCDX2 and CX3). (**C**) The RAD51 nucleoprotein filament invades the homologous template in search for a homologous sequence. The strand invasion by the RAD51 filament forms a D-loop structure. RAD54 aids in these processes. (**D**) RAD51 is displaced and the DNA is extended by polymerases that copy the missing nucleotides from the repair template. (**E**) The second end of the DSB is captured and the DNA intermediate is resolved through resolution or dissolution, resulting in either a crossover or non-crossover product. HR resolution is aided by MUS81–EME1, GEN1 or SLX1/4, whereas dissolution occurs through the action of BLM–TOPIIIα–RMI1/2. (**F**) RAD51 functions at stalled replication forks. When the replication fork encounters a fork-blocking lesion (yellow starburst), RAD51 promotes replication fork reversal and protects the nascent strands of DNA from degradation by exonucleases (pink pac-man).

The stability of our genome is continually threatened by both endogenous and exogenous sources of DNA damage (10). Repair of DNA damage is central to preventing the development of cancer. While many types of DNA damage can occur, the most toxic DNA lesion is a DNA double-strand break (DSB) (11). One high-fidelity DSB repair mechanism is HR (Figure 1) (12). HR is considered error-free because it uses a homologous template for repair to restore any missing nucleotides at the break site (12). After DSB formation, the DNA ends are resected and coated by the single-stranded DNA (ssDNA)-binding heterotrimer replication protein A (RPA), consisting of RFA1, RFA2 and RFA3 (Figure 1A) (13). Subsequently, RPA becomes displaced by RAD51, which forms a nucleoprotein filament of RAD51 protomers around the ssDNA end (Figure 1B) (14). The RAD51-coated ssDNA performs the homology search and subsequent strand invasion steps that define HR (Figure 1C) (15). RAD51 strand invasion of a homologous template forms a displacement loop (D-loop) structure (Figure 1C) (15). Once the homologous sequence is invaded, RAD51 is displaced, allowing polymerases to replicate the homologous template (Figure 1D) (16). The second end of the DSB can then be captured, and these joint DNA molecules are resolved with the assistance of many different enzymes (i.e. nucleases, helicases, topoisomerases) (16). Ultimately, resolution or dissolution of these DSB repair intermediates results in crossover or non-crossover products (Figure 1E) (15).

## RAD51 AND THE DNA REPLICATION STRESS RESPONSE

Besides its role in DSB repair, RAD51 is crucial for several DNA transactions at stalled or collapsed replication forks during the replication stress response (Figure 1F). First, RAD51 promotes replication fork reversal, which occurs when the fork encounters a replication block and reverses direction to continue replication (17,18). The mechanism by which RAD51 promotes this reversal is an active area of investigation (19,20). Second, RAD51 protects the nascent strands of DNA from degradation by exonucleases at both stalled and reversed replication forks (21,22). By blocking nuclease activity at stalled forks and DSBs, RAD51 prevents degraded fragments of DNA from triggering the STING-mediated innate immune response (23). Third, RAD51 is involved in the restart of forks during replication blocks, through its protection and reversal activities (24). Additionally, RAD51 and HR are key players during the repair and tolerance of DNA cross-links (25). It remains to be determined how the different roles of RAD51 during HR and replication uniquely contribute to cancer.

## REGULATION OF RAD51

RAD51 activity is regulated by proteins that promote RAD51 assembly on ssDNA ends or disassembly of ectopic RAD51 filaments and after RAD51-mediated homology search, such as RAD54 (6,7,14). In mammalian cells, positive regulators of RAD51 include BRCA2, PALB2 and the RAD51 paralogs (6). PALB2 recruits BRCA2 to ssDNA (26). In addition to recruiting BRCA2, PALB2 also binds to both DNA and RAD51; these interactions enhance strand invasion activity (27,28). BRCA2 binds to RAD51 and stimulates assembly of RAD51 protomers onto ssDNA to form the nucleoprotein filament (29,30). Upon nucleation of the RAD51 filament, the RAD51 paralogs (including RAD51B, RAD51C, RAD51D, XRCC2, XRCC3 and SWSAP1) help to stabilize and elongate the RAD51 filament (7,31). The RAD51 paralogs arose from early gene duplications of the RAD51 ancestor, archaeal RadA, and therefore share a similar sequence to RAD51 itself (32). The precise functions of the RAD51 paralogs have yet to be fully elucidated (7).

Additionally, RAD51 expression is regulated by several transcription factors as well as post-translational modifications via phosphorylation. p53 is a transcriptional regulator of RAD51 and represses RAD51 protein and mRNA expression (33,34). Furthermore, in soft tissue sarcoma cell lines, transcriptional repression of RAD51 via p53 is mediated by activator protein 2 (34). Conversely, positive regulation of RAD51 expression in cancerous cells is mediated by the EGR1 transcription factor (35). Post-translational modification of RAD51 by phosphorylation promotes its repair activities (36–39). For example, the receptor tyrosine kinase c-MET phosphorylates several tyrosine residues on RAD51, which increases the stability of the presynaptic filament (37). Polo-like kinase 1 also phosphorylates RAD51, which enables casein kinase 2 to phosphorylate RAD51 on threonine 13 (36). This triggers the binding of RAD51 to Nijmegen breakage syndrome gene product (NBS1) (36). The binding of RAD51 to NBS1 is critical for cellular resistance to genotoxic stress (36).

## RAD51 AND GENOME STABILITY

Given the importance of RAD51 in HR and replication stress response, it is not surprising that the accurate regulation of RAD51 activity is critical to preserve genome stability. The formation of the RAD51 nucleoprotein filament on ssDNA is central to all RAD51 functions and the commitment to HR. ssDNA occurs in many different scenarios such as during DNA replication or as a repair intermediate for other DNA damage pathways. Therefore, regulating RAD51 assembly on ssDNA at the right time and place is critical. RAD51 binding to ssDNA, and consequently the assembly and disassembly of the RAD51 filament, is dependent on RAD51 binding, the RAD51 protein levels and the activity of both positive and negative regulators of HR. Underscoring the importance of tightly controlling RAD51 activity, upregulation or downregulation is associated with genome instability and cancer (8,40).

Inactivating *RAD51* can have profound effects on genome stability leading to cancer as well as an FA-like syndrome, a rare genetic disorder characterized by bone marrow failure and cancer (6,7,41). *RAD51* activity can be disrupted by direct mutations in the *RAD51* gene or through alterations in its regulators such as *BRCA2*, *PALB2* and the *RAD51* paralogs (6,41). While *RAD51* mutations are associated with many cancer types, mutations in genes that regulate *RAD51* are more closely associated with breast and ovarian cancers (6,7,42,43). Additionally, decreased RAD51 expression has also been observed in certain cancers, particularly sporadic breast cancers (44). Defects in HR result in a unique mutation signature, termed ‘Signature 3’, and can be used as a marker for determining therapeutic response to specific chemotherapeutic agents (45). For example, ovarian cancers harboring inactivating *BRCA2* mutations can be specifically treated with poly(ADP-ribose) polymerase (PARP) inhibitors (PARPi) (46,47). PARP inhibition also has the potential to treat other non-BRCA HR-deficient tumors. There are several commercially available HR repair deficiency (HRD) assays that can be used in the clinic that examine tumor samples using biomarkers such loss of heterozygosity (LOH) (48). Despite these advances, resistance mechanisms to PARPi occur, which has led to the current efforts to make PARPi more efficacious.

While cells that overexpress RAD51 are resistant to DNA-damaging agents, including radiation and cisplatin, RAD51 overexpression also causes aberrant and excessive recombination, which promotes genome instability and is observed in many cancer types (8,44). As a result, compounds that alter RAD51 activity are being developed as novel cancer therapeutic targets (49). These strategies range from small molecule inhibitors to antibodies. In this review, we will focus on how misregulation of RAD51, both upregulation and downregulation, results in FA-like syndrome and cancer predisposition. We will also discuss the rationale behind current clinical treatments and new therapeutic approaches in development.

## MUTATIONS IN *RAD51* AND ITS GENE FAMILY IN HUMAN DISEASE

### RAD51 gene family mutations and FA-like syndrome

FA and FA-like patients with mutations in RAD51 and the RAD51 paralogs have been identified (50). FA is a rare genetic disease known to affect numerous systems throughout the human body, including the skeletal and immune systems (50). The most notable symptoms of FA include severe bone marrow failure, skeletal defects, premature aging and an unusually high predisposition to a variety of cancers (50). Patients with FA have an increased risk for acute myeloid leukemia and squamous cell carcinoma as well as aggressive solid tumors, particularly of the head and neck, which are prevalent during adolescence (51). Unfortunately, the most effective treatment for FA, a bone marrow transplant, also leads to the increase in malignancies commonly seen in patients’ later years (50). FA is often difficult to diagnose due to the wide array of symptoms and similarities to other genetic disorders (51). To correctly diagnose FA, interstrand cross-linking (ICL) agents such as mitomycin C (MMC) or diepoxybutane are used to test the repair ability of the cells (50,51). ICL repair is accomplished by the FA pathway, which consists of 22 gene products (FANCA-W), many of which are involved in ICL repair and other DNA repair pathways (50–52).

Notably, many of the FA genes include the *RAD51* gene family and to date have been identified as follows: *FANCO* (*RAD51C*), *FANCR* (*RAD51*) and *FANCU* (*XRCC2*) (51). In addition, proteins that regulate RAD51 have also been identified as FA genes such as *BRCA2* (*FANCD1*) and *PALB2* (*FANCN*). An interesting RAD51 mutation, RAD51-T131P, was found in a patient displaying an FA-like phenotype and these cells exhibit high sensitivity to cross-linking agents while still demonstrating HR proficiency (53). Therefore, this RAD51-T131P point mutation defines a separation-of-function allele that uncouples RAD51 function during HR from ICL repair (53). In addition to *RAD51* mutations, another patient displaying a typical FA phenotype was found to have a novel truncating mutation in the *RAD51* paralog, *XRCC2* (54). This discovery led to reclassification of *XRCC2* as a bona fide FA gene, *FANCU* (*XRCC2*) (54). Mutations in FA genes are also associated with cancer development in individuals who do not have FA (55). While biallelic mutations in one of the FA genes can give rise to FA, heterozygous mutations in many of these same genes, including *RAD51C* and *BRCA2*, result in cancer predisposition (51). A prime example of this is *RAD51C* or *FANCO*. A biallelic *RAD51C* point mutation in residue R258H was found in a patient who exhibited FA-like symptoms, while monoallelic mutations in RAD51C are observed in hereditary breast and ovarian cancers (7,56,57). It should be noted that although *RAD51C* is included in the list of FA genes it is actually considered an FA-like gene because it does not exhibit all of the classic FA phenotypes (52).

### Breast/ovarian cancer predisposition

**Figure 2.**
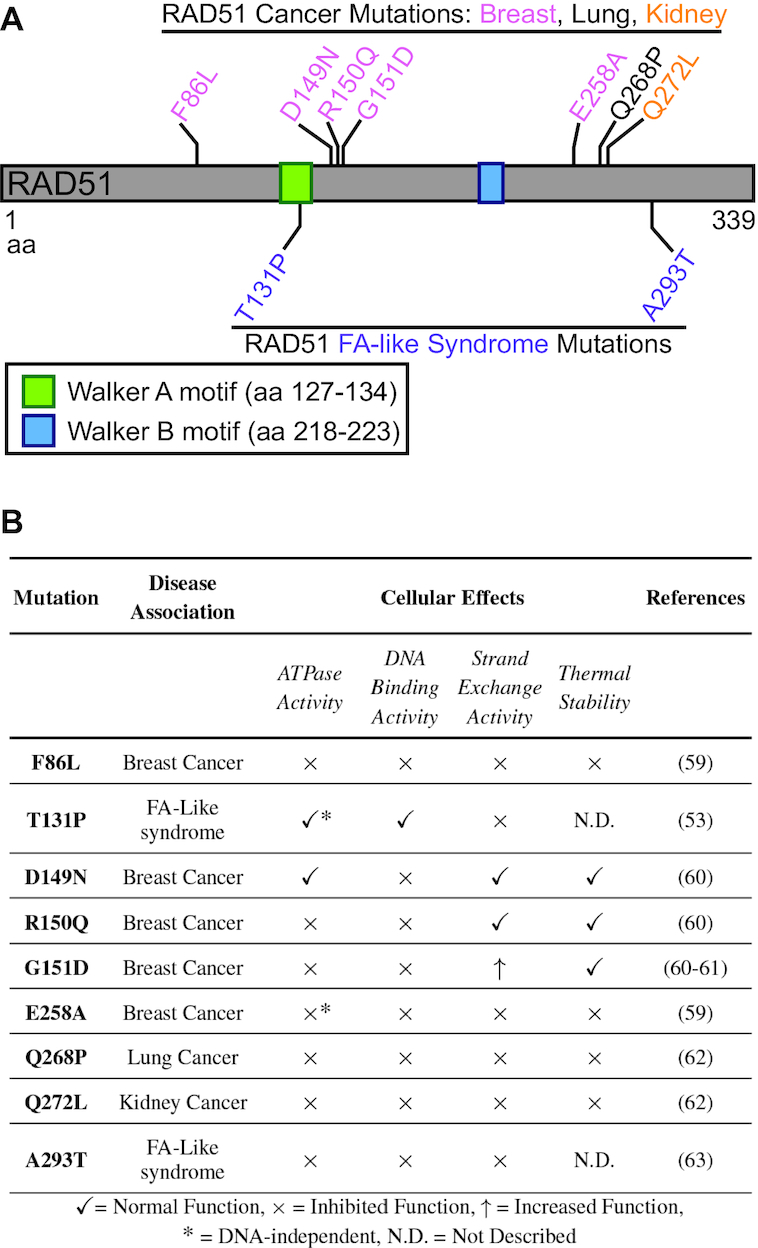
(**A**) Schematic of the RAD51 protein showing the Walker A and B motifs and the functionally analyzed disease-associated missense mutations. RAD51 is 339 amino acids (aa) long with Walker A and B motifs (green and blue boxes, respectively). Breast, lung and kidney cancer-associated mutations are shown in pink, black and orange, respectively. FA-like syndrome-associated mutations are shown in purple. (**B**) Table shows a list of the functionally analyzed RAD51 mutations that are associated with cancer or FA-like syndrome. Each mutation has been investigated for its effects on RAD51 in regard to its ATPase activity, DNA binding activity, strand exchange activity and thermal stability. A check mark indicates normal RAD51 function, an ‘x’ indicates inhibited RAD51 function, an up arrow indicates increased RAD51 function and a star indicates that ATPase activity is independent of the addition of ssDNA, unlike WT RAD51. Note that F86L, D149N, G151D, Q268P and Q272L are somatic mutations, whereas T131P, R150Q, E258A and A293T are germline mutations.

Mutations in *RAD51* and its regulators are strongly associated with genome instability and cancer predisposition (6,7,58). Because the RAD51 nucleoprotein filament is so critical for HR, its mutation can detrimentally threaten the integrity of the genome (44). Although over 90 cancer- and FA-associated missense variants in *RAD51* have been identified, only a handful of these mutations have been functionally characterized (Figure 2B). Deleterious disease-associated *RAD51* variants are found to inhibit RAD51 ATPase activity (i.e. F86L, R150Q, G151D, E258A, Q268P, Q272L, A293T), DNA binding (i.e. F86L, D149N, R150Q, G151D, E258A, Q268P, Q272L, A293T), strand exchange (i.e. F86L, T131P, E258A, Q268P, Q272L, A293T) and/or thermal stability (F86L, E258A, Q268P, Q272L) (Figure 2) (53,59,60). Importantly, some of these variants can uncouple the different RAD51 activities suggesting that each of these activities is independently critical for genome stability. For example, monoallelic germline *RAD51* mutations T131P and A293T, located in RAD51’s ATPase domain, confer an FA-like phenotype (53,63). While both RAD51-T131P and RAD51-A293T mutant proteins exhibit impaired DNA strand exchange, only RAD51-A293T diminishes DNA binding and ATPase activities (53,63). Additionally, greatly reduced strand exchange activity is observed when several of these variants are mixed with wild-type (WT) RAD51, suggesting that some of these variants are dominant mutations (59–60,62). Therefore, these RAD51 variants may be producing a dominant negative effect by poisoning WT RAD51 function (59).

Additional RAD51 variants confer an increased risk of breast, endometrial and prostate cancer (42,64–68). For example, *RAD51C*-G135C is associated with an increased risk of endometrial cancer [RAD51-G135C, C/C: odds ratio (OR) 3.72 (95% confidence interval (CI) 2.77–5.00), C: OR 2.54 (95% CI 2.16–2.99)] and breast cancer [RAD51-G135C, allele model: OR 4.32 (95% CI 2.63–7.10), dominant model: OR 2.28 (95% CI 1.44–3.60), recessive model: OR 10.27 (95% CI 14.71–22.38), homozygous model: OR 7.26 (95% CI 3.59–14.68)] (65,66). However, functional analysis of this variant has not been performed to date. Furthermore, the same RAD51 variant, *RAD51C*-G135C, also increases the risk for hematologic malignancies [RAD51-G135C, C versus G: OR 1.16 (95% CI 1.02–1.31), dominant model: OR 1.18 (95% CI 1.03–1.36)] (69).

HR deficiency caused by mutations or promoter methylation in HR genes is found in ∼50% of hereditary ovarian tumors (70–72). In addition to inactivating pathogenic mutations in *RAD51*, disruption of the *RAD51* regulators, including the *RAD51* paralogs, is also correlated with cancer (56,71,73). *RAD51* and its regulators can be interrupted via missense mutations, promoter methylation, copy number changes, truncations and deletions (70,71,74). Patients with pathogenic mutations in these *RAD51* regulator genes, particularly *RAD51C* and *RAD51D*, have a higher risk of developing ovarian cancer [RAD51C, OR 8.3 (95% CI 5.43–12.48); RAD51D, OR 3.17 (95% CI 1.31–7.42)] (75). In ovarian cancer, monoallelic *RAD51C* and *RAD51D* mutations are primarily germline, although somatic mutations have also been identified (71,76). It is estimated that the frequency of *RAD51C* and *RAD51D* germline mutations in ovarian cancer patients is 3% and 5%, respectively (71). These monoallelic germline *RAD51C* and *RAD51D* mutations become homozygous in the tumor likely due to LOH events (77–79). *RAD51C* and *RAD51D* are the most frequently mutated RAD51 paralogs in cancers and are most closely associated with increased ovarian cancer risk, although breast cancer risk is also increased (75,80). A recent study of multiple hereditary cancer genes identified both *RAD51C* [OR 1.84 (95% CI 1.28–2.71)] and *RAD51D* [OR 2.09 (95% CI 1.2–3.72)] as having an elevated risk of breast cancer (75). Additionally, there is also an increased breast cancer risk associated with the *BRCA2* [OR 4.86 (95% CI 4.11–5.74)] and *PALB2* [OR 5.1 (95% CI 4.06–6.4)] genes (75). Therefore, *RAD51C* and *RAD51D* genes are included in several hereditary breast/ovarian cancer screening panels (81).

In contrast to *RAD51C* and *RAD51D*, *XRCC2*, *XRCC3* and *RAD51B* variants are less frequently observed in tumors and the cancer risk for individuals harboring these variants remains controversial (82–85). In part, this may be due to insufficient number of patients with known pathogenic variants to accurately determine cancer risk. Additionally, for the RAD51 paralog variants that have been identified, very few have been functionally analyzed. The cancer risk for most of these variants is unknown and therefore they remain classified as variants of unknown significance (VUS). The lack of functional analysis for these VUS is clinically challenging (7). HR proficiency, or lack thereof, is a good measure of the potential pathogenicity of a variant and can also be used to identify tumors that would benefit from PARPi (86).

### Identifying HR deficiency in patient tumors

Determining HR deficiency in the absence of a *BRCA1/2* mutation is critical since HR status correlates with a therapeutic response. Patients with HR-deficient tumors demonstrate a better response to both traditional chemotherapy and PARPi (87). The development of distinct biomarkers that identify HR deficiency in tumors is an area of active investigation. For example, germline BRCA mutations, platinum sensitivity and HRD assays are currently used to determine HR deficiency in patient tumors and subsequent PARPi use (88). Novel methods in development to identify HR deficiencies include examining the mutational profile of HR genes, identifying ‘genomic scars’ caused by deficient HR and assessing dynamic HR markers in real time (88). Genomic scar assays identify distinct genomic abnormalities left behind by deficient HR, including specific mutation patterns (Signature 3) and LOH (88). In addition, several novel assays to determine the HR status of cells have centered on RAD51 foci formation (89–91). RAD51 foci are effective in predicting which patient breast tumor samples will be responsive to traditional chemotherapy or PARPi (92,93). Additionally, the presence of RAD51 foci in germline *BRCA*-proficient breast cancers correlates with resistance to PARPi (94). Therefore, RAD51 foci could be used as a reliable marker to help identify which patients would benefit most from PARPi (93,94). However, the use of RAD51 foci as a biomarker is not without challenges. The assay used to identify RAD51 foci is technically difficult and furthermore the foci need to be induced with DNA damage (95). As an alternative approach, patient-derived organoids are being used to assess DNA repair ability and sensitivity to therapeutics (96–99). Because the organoid is derived from an individual’s tumor, organoids provide a personalized approach to cancer therapeutics and drug discovery (99). Although larger patient numbers are needed to further assess the efficacy of these assays, the use of organoids in cancer research has thus far shown promising results. For example, organoid lines retain similar molecular and mutational profiles as the parental tumors from which they were derived (97,98). In many cases, the organoid’s response to a particular drug therapy was analogous to the response seen in the patient after treatment with the same therapeutic (96). Since HR deficiency is indicative of which therapeutic strategies will be most effective, both RAD51 foci and organoid models enable screening to better predict patients who will respond to treatment.

### Treatment of HR-deficient tumors and mechanisms of resistance

Mutations in *BRCA1/2* ultimately lead to HR deficiency in part due to an inability to form RAD51 filaments. Unlike RAD51 and its other regulators, *BRCA1/2* mutation carriers are more prevalent and individuals with these known pathogenic variants are the most studied (58). For example, *BRCA1* and *BRCA2* are mutated in ∼15% of ovarian cancers and ∼6% of breast cancers (100,101). In some populations, *BRCA* mutations are more frequently observed such as in Ashkenazi Jews with a carrier frequency of 1 in 40 (102). Therefore, it is important to consider how *BRCA1/2*-deficient tumors are being treated as the vast majority of patients currently targeted with a synthetic lethal approach have *BRCA*-mutated cancers.

While HR defects promote cancer development, these deficiencies also provide an optimal therapeutic target that can be exploited through synthetic lethality (SL). This phenomenon results from two independent but overlapping pathways that when disrupted individually are viable but when disrupted together result in cell death (103,104). Often, multiple DNA repair pathways can process the same DNA lesion that provides a window of opportunity to target synthetic lethal gene combinations (10). For example, loss of a high-fidelity DNA repair pathway can result in increased mutational burden if the alternative repair pathway is mutagenic (105,106). Therefore, cancer cells that become reliant upon an alternative repair pathway provide a unique therapeutic window to damage only the tumor through SL (106,107). Similarly, SL can also occur by pharmacological inhibition of a gene or pathway that has a synthetic lethal interaction with a mutation found in the tumor (106). In contrast to traditional chemotherapeutic treatments that cause indiscriminate DNA damage to all cells, the synthetic lethal approach enables selective cell killing in the tumor (72,106–108). One of the first monotherapies to successfully exploit SL in a clinical setting is PARPi.

PARPi are small molecule drugs that target PARP, a crucial enzyme in the repair of single-strand breaks (109,110). An incredible discovery was made when *BRCA1/2*-deficient cells were found to be sensitive to PARPi (46,47,111). The sensitivity of *BRCA*-deficient cells to PARPi is directly due to HR loss (112). While PARPi alone does not result in cell lethality and is less cytotoxic to normal cells, adverse effects have been reported (113). It is important that LOH in the *BRCA1/2* genes occurs within the tumor itself, but not the surrounding tissue and is an enabling characteristic of cancer development (46,47,79). Therefore, combining *BRCA* loss with PARP inhibition results in cell lethality in the tumor but not the remaining tissue. Thus, other HR-deficient tumors may also benefit from PARP inhibition.

PARPi prevent NAD^+^, the substrate required for enzymatic activity, from binding to PARP, which results in the trapping of PARP on the DNA (114,115). If PARP is unable to disengage from the ssDNA, this results in stalled or collapsed replication forks that subsequently form DSBs (114). In cells with impaired HR, alternative DNA repair pathways that are more mutagenic are needed to repair the damage, which leads to accumulation of errors and eventual cell death (105,106). Recently, an alternative model for PARP function has been proposed suggesting that PARP facilitates the ligation of Okazaki fragments during replication (116). PARP inhibition results in PARP becoming trapped on DNA containing unligated Okazaki fragments (116). In this scenario, HR may be required to remove these unligated Okazaki fragments, which enables PARPi to specifically target HR-deficient cells (116). It is likely that a combination of these activities is required for the efficacy of PARPi upon HR deficiency.

To date, there are four PARPi currently approved by the FDA to treat breast, ovarian, Fallopian tube, prostate and primary peritoneal cancers: rucaparib (Rubraca), olaparib (Lynparza), niraparib (Zejula) and talazoparib (Talzenna) (117). One other PARPi in clinical development is veliparib (ABT-888); however, it has yet to be approved by any agency (118). Talazoparib and veliparib have the strongest and weakest PARP trapping ability, respectively, while olaparib, rucaparib and niraparib are considered medium trappers (119). Initially, PARPi were approved as a monotherapy for ovarian cancer patients with germline *BRCA* mutations that have been treated with three or more chemotherapeutics (117). However, since their emergence in 2014, PARPi have been approved to treat a wider range of cancer indications (117). In 2017, olaparib received approval by the FDA as an adjuvant maintenance therapy in epithelial ovarian, Fallopian tube and primary peritoneal cancers that respond to platinum-based therapies (117). In a more recent report, results from a landmark clinical trial revealed significantly increased overall survival (12.9 months) in ovarian cancer patients treated with olaparib as an adjuvant therapy (120). PARPi are also effective against other *BRCA1/2*-deficient tumor types such as pancreatic and prostate cancers (121–123). There are currently clinical trials underway that are investigating the efficacy of PARPi in multiple cancer types, including those without *BRCA1/2* mutations, which might have mutations in other HR genes (124).

### HR proteins and PARPi resistance

**Figure 3.**
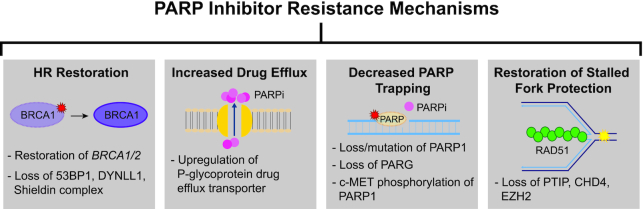
PARPi inhibitor resistance mechanisms. While HR-deficient tumors can be treated with PARP inhibitors, high rates of PARPi-resistant cells are observed in the clinic and can lead to the proliferation of PARPi-resistant tumors. PARP inhibitor resistance occurs by (i) restoration of HR (represented by genetically restoring BRCA1 function), (ii) increased drug efflux (represented by a drug efflux pump in yellow and PARPi in pink), (iii) decreased PARP trapping (represented by PARP mutation/loss in a dotted circle and PARPi in pink) and (iv) restoration of stalled fork protection (represented by a replication fork in blue and a green RAD51 filament). Examples of each mechanism are shown in the gray boxes as described.

Although PARPi are now widely used for HR-deficient tumors, PARPi resistance is a complication frequently observed in the clinic. Cancer cells acquire resistance by multiple mechanisms such as HR restoration, restoration of replication fork protection activities, drug efflux increases or disruption of PARP-related activities (Figure 3) (112,125). However, decreased PARP trapping via mutation of *PARP1* and restoration of HR gene function (reversion mutations) are the only mechanisms that have been observed in tumors to date (112).

Similar to platinum therapy, recovering functional *BRCA1/2* genes through a reversion mutation is a driving mechanism behind PARPi resistance (126,127). More recently, PARPi resistance via reversion mutations has also been observed in other HR genes, including *RAD51C* and *RAD51D* (128). In addition to reversion mutations in the HR genes themselves, disruption of other genes leading to HR restoration has also been observed in cell culture models. For example, in *BRCA1*-deficient cells, HR restoration is accomplished through loss of *TP53BP1*, the Shieldin complex or *DYNLL1*, among others (129–134). *BRCA1/2*-deficient cells can also become resistant to PARPi through loss of the several HR repair-related proteins, including the Pax transactivation domain-interacting protein (PTIP), CHD4 and EZH2 (135–137). Unlike the other genes that directly restore HR, PTIP, CHD4 and EZH2 loss results in increased fork protection from nucleases such as MRE11 and MUS81 (135–137).

Tumor cells can also increase the removal of PARPi from the cell by upregulating the expression of genes encoding P-glycoprotein efflux pumps, which results in diminished intracellular availability (138–140). Additionally, cells may acquire PARPi resistance by decreased PARP trapping via mutations in *PARP1* or loss of *PARG* (112,141,142). PARPi resistance can also occur by PARP1 phosphorylation on tyrosine 907 by c-Met, which increases the enzymatic activity of PARP1 while decreasing binding to the PARPi (143,144). Furthermore, cancer stem cells are resistant to PARPi and exhibit increased RAD51 foci formation after DNA damage (145). The combination of PARPi and RAD51 inhibition could be useful in overcoming PARPi resistance (145). Due to these resistance mechanisms, the addition of PARPi in the clinic has resulted in modest increases in progression-free survival for ovarian cancer patients (5–8 months depending upon the inhibitor used) (118). There are inherent challenges involved with the use of PARPi, but even so they have revolutionized the way in which we treat *BRCA*-deficient cancers and initiated investigations into other potential HR inhibitors.

## RAD51 AS A THERAPEUTIC TARGET IN CANCER TREATMENT

### Optimal RAD51 expression levels are required for normal cellular function

While the overexpression of RAD51 is common in many cancers, downregulation of RAD51 has also been reported. Insufficient RAD51 levels lead to unrepaired DNA damage and genome instability that predispose cells to cancer. Decreased expression of RAD51 is observed in ∼28% of non-hereditary breast cancers and several classes of renal cancer carcinomas, including clear cell and papillary (146,147). RAD51 expression levels in breast cancers can vary depending on *BRCA1* status and hormone receptor expression (148). Estrogen receptor and *BRCA1*-negative sporadic breast cancers exhibit low levels of nuclear RAD51, which is associated with poor prognosis (92,148). It has been suggested that the hypoxic condition of the tumor microenvironment can also cause decreased RAD51 expression resulting in HR deficiency (149–151). Hypoxia is a common condition observed in solid tumors (152). These results suggest that RAD51 protein levels can be indirectly affected even without a corresponding genetic mutation.

Comparatively, RAD51 overexpression is also observed in numerous cancers, including pancreatic, melanoma, breast, non-small cell lung, prostate and glioblastoma (153). RAD51 overexpression occurs by excessive RAD51 promoter activation in which promoter activity is upward of 840-fold compared to normal cells (154). The RAD51 promoter can be stimulated by multiple oncogenes that gradually increase its activity as cells progress toward malignancy (35). Cancer patients who exhibit high RAD51 expression have lower overall survival rates and poor clinical outcomes (153,155). For example, evaluating cancer patient survival relative to RAD51 expression indicates that high RAD51 expression in breast and liver cancers correlates with a lower survival probability (156). When RAD51 is overexpressed in cancer cells, there is a noticeable increase in HR activity, and this change can enable resistance to traditional cancer therapies (8). Therefore, making HR-proficient tumor cells HR-deficient by inhibiting RAD51 may increase the effectiveness of current therapies. Current therapies include PARPi, which are used to treat HR-deficient tumors (109). Additionally, RAD51 inhibitors could prove useful in restoring SL in tumors that have developed PARPi resistance. Accordingly, there are a number of groups developing RAD51 inhibitors in order to further exploit the HR pathway as a therapeutic target. The inhibitors we will discuss here are listed in Table 1.

**Table 1.** Novel modulators of RAD51 in development

**Compound**	**Cellular effects**	**References**
*Small molecules*		
**DIDS**	*Increased*: RAD51-mediated ATP hydrolysis	(163)
	*Decreased*: RAD51-mediated strand exchange; RAD51 homologous pairing	
**B02**	*Increased*: Doxorubicin, MMC, cisplatin and PARP1 inhibitor sensitivity; cell death	(164–167)
	*Decreased*: HR; IR-induced RAD51 foci formation; D-loop formation	
**OA-NO_2_**	*Increased*: Doxorubicin, olaparib, cisplatin and IR sensitivity	(168)
	*Decreased*: HR; IR-induced RAD51 foci formation	
**Chicago Sky Blue**	*Decreased*: IR-induced RAD51 foci formation; RAD51 homologous pairing	(169)
**Halenaquinone**	*Decreased*: IR-induced RAD51 foci formation; RAD51 homologous pairing	(172)
**RI(dl)-1**	*Decreased*: HR; D-loop formation	(173)
**RI(dl)-2**	*Increased*: IR sensitivity	(173)
	*Decreased*: HR; D-loop formation	
**RI-1**	*Increased*: MMC sensitivity	(174)
	*Decreased*: HR; MMC-induced RAD51 foci formation	
**RI-2**	*Increased*: MMC sensitivity	(175)
	*Decreased*: HR	
**IBR2**	*Increased*: Receptor tyrosine kinase, microtubule inhibitor sensitivity	(177,178)
	*Decreased*: HR; IR-induced RAD51 foci formation; RAD51 protein levels	
**BRC peptide**	*Decreased*: RAD51-mediated strand exchange	(179)
**RS-1**	*Increased*: HR; D-loop formation; cisplatin resistance; toxic RAD51–DNA complexes	(180,181)
**CYT-0851**	*Increased*: Cell death; tumor growth delay	NCT03997968
*Antibodies*		
**3E10**	*Increased*: IR, doxorubicin, ATR inhibitor sensitivity	(183–186,189)
	*Decreased*: HR; RAD51-mediated strand exchange; RAD51 foci formation and nuclear localization	
**Fab-F2-iPTD**	*Increased*: MMS sensitivity	(187)
	*Decreased*: Cellular growth	

List of RAD51 modulators in development and their cellular effects. The cellular effects of each modulator are split into two categories: one for outcomes that lead to increased reactions and the other for decreased reactions.

### Novel therapies that modulate RAD51

**Figure 4.**
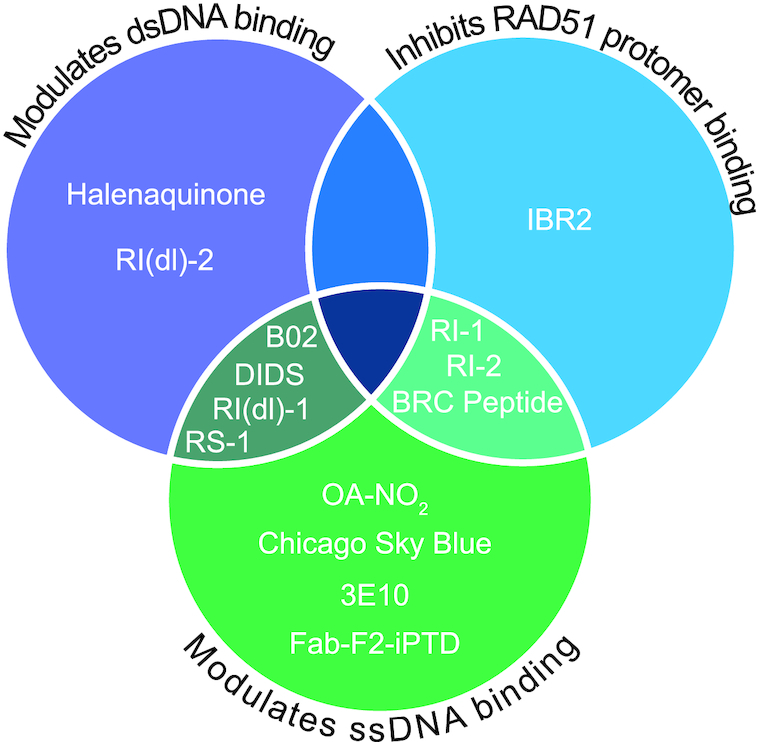
Venn diagram showing the mechanisms by which each RAD51 modulator alters RAD51–dsDNA binding (purple circle), RAD51–ssDNA binding (green circle) and RAD51 protomer–protomer binding (blue circle). Note that several of the drugs inhibit multiple binding mechanisms as indicated in the overlapping circle areas. Note that CYT-0851 is not included in the diagram as its mechanism of action has yet to be disclosed.

These inhibitors and stimulators function by modulating the binding of RAD51 to ssDNA and/or dsDNA or by inhibiting RAD51 protomer–protomer interactions (Figure 4) (157,158). The resulting mechanisms of action lead to altered nucleoprotein filament and/or D-loop formation (157,158).

#### Small molecule modulators of RAD51

Early forays into RAD51 inhibition began with the small molecule inhibitor amuvatinib (MP-470), which was originally developed to target multiple tyrosine kinases (159). Amuvatinib was found to reduce expression of RAD51 and increase DSBs in glioblastoma multiforme cell lines upon treatment with ionizing radiation (IR) (160). Although amuvatinib decreased RAD51 expression in a dose-dependent manner, the mechanism by which this occurs is unknown, and thus it may or may not directly target RAD51 (159). Amuvatinib phase I clinical trials resulted in reduced RAD51 expression in many patient tumors similar to *in vitro* studies using human cancer cell lines (161). However, a subsequent phase II trial with amuvatinib in conjunction with cisplatin or carboplatin did not result in abatement of RAD51 expression, and therefore clinical development of this drug was suspended (162). While RAD51 inhibitors are a promising target, preclinical investigation is still needed to better understand RAD51 targeting and develop more effective compounds.

DIDS prevents RAD51–ssDNA and RAD51–dsDNA binding by attaching to RAD51 directly (163). However, the elevated toxicity of DIDS on cultured human cells has hindered its development (163). B02 is a compound that was identified as a high-specificity RAD51 inhibitor that directly binds to RAD51 (164,165). B02 impedes HR by preventing RAD51 from binding to both ssDNA and dsDNA, which further sensitizes cells to DNA-damaging agents such as cisplatin and MMC (164–166). Multiple myeloma cells treated with B02 in conjunction with the topoisomerase II inhibitor doxorubicin exhibited enhanced cell death through DSB induction and subsequent blocking of HR repair (167). Recently, the fatty acid nitroalkene OA-NO_2_ was found to target RAD51 and increase the effects of doxorubicin, olaparib, IR or cisplatin in triple-negative breast cancer cell lines (168). When OA-NO_2_ alkylates RAD51 at cysteine 319, it prevents RAD51 from binding to ssDNA, and thus decreases HR (168). Chicago Sky Blue is another compound that inhibits RAD51–ssDNA binding and strand exchange activities (169). However, the mechanism by which this inhibition occurs is still unknown (169).

The aforementioned inhibitors interfere with RAD51’s role in both HR and replication fork protection. Unlike in HR, RAD51 replication fork protection functions only require ssDNA binding activity (170). There are a few compounds that impair RAD51 strand exchange activity, while preserving its ssDNA binding, and therefore its replication fork-associated functions (171). An extensive screen of marine sponge extracts revealed a natural compound, halenaquinone, that inhibits RAD51 by preventing the RAD51–ssDNA filament from forming a D-loop with its homologous dsDNA substrate (172). Furthermore, halenaquinone-treated cells exhibit significantly reduced IR-induced RAD51 foci (172). However, while halenaquinone directly binds to RAD51, it does not alter RAD51 affinity for ssDNA (172). Like halenaquinone, a high-throughput screen identified two additional novel RAD51 inhibitors, RI(dl)-1 and its analog RI(dl)-2, that specifically inhibit D-loop activity (173). While RI(dl)-1 minimally inhibits ssDNA binding, RI(dl)-2 has no effects on ssDNA binding while still blocking D-loop formation (173). Additionally, RI(dl)-2 significantly impairs HR in human cells and sensitizes multiple cancer cell lines to IR (173).

In addition to inhibitors that target RAD51–ssDNA and/or RAD51–dsDNA binding, RAD51 inhibition can also occur by blocking its protein interaction with itself. RI-1 was discovered in a high-throughput screen for RAD51 inhibitors (174). RI-1 and its analog RI-2 block RAD51 protomer–protomer interactions by binding cysteine 319, which is located in the protomer interface (174–176). RI-1 binding to RAD51 prevents RAD51 filament formation and subsequent D-loop activities (174). RI-1 also increases the sensitivity of cancer cells to MMC (174). The RI-1 analog, RI-2, was developed to limit the off-target effects of RI-1 and to achieve a longer half-life (175). RI-2 displays the same RAD51 inhibitory effect as RI-1 and can still sensitize cells to MMC. However, RI-2’s bond to RAD51 is reversible, thus increasing the stability of the compound (175). Another small molecule that directly binds RAD51 is IBR2 (177). Once bound, IBR2 prevents RAD51 protomer–protomer binding and inhibits growth in multiple cancer cell lines (177,178). Lastly, RAD51 filament formation can also be inhibited by a short BRC-motif peptide derived from BRCA2 that selectively binds the protomer–protomer interface of RAD51 (179). This interaction also prevents RAD51 from binding to ssDNA, thus inhibiting filament formation and strand exchange activities (179).

Since underexpression of RAD51 can lead to unrepaired DNA damage and thus threaten the stability of the genome, upregulation of RAD51 activity is also being exploited as a potential therapy. The small molecule RS-1 was developed to stimulate binding of RAD51 to ssDNA and dsDNA, independent of ATP hydrolysis (180,181). While cancer cells often rely on high RAD51 expression levels to subvert DNA-damaging agents, RAD51 overexpression can also result in deleterious recombination events, even on undamaged DNA (182). RS-1 induces genotoxic RAD51 recombination in cancer cells, which have more ssDNA due to increased replication, that leads to cell death while having minimal effect on untransformed cells (181). This selective cell killing can be increased by disrupting the two RAD54 translocases, specifically RAD54L and RAD54B, that normally help remove RAD51 during HR (181).

#### Antibody inhibitors of RAD51

The Glazer group discovered an autoantibody to RAD51 called 3E10 (183), which was originally identified as an autoantibody associated with lupus and is considered benign to noncancerous cells (184). 3E10 directly interacts with the N-terminus of RAD51, and this interaction prevents RAD51 from assembling as a filament onto ssDNA (183). An expanded role for 3E10 was demonstrated as 3E10 exposure results in increased toxicity in PTEN-deficient glioma and melanoma cancer cells, which already have a significant propensity for DNA damage (185). Additionally, 3E10 sensitizes tumor cells to various cancer therapies, including radiation, doxorubicin and ATR inhibitors (185,186). Recently, a novel RAD51 inhibitor, Fab-F2-iPTD, was created by fusing a cell-penetrating peptide to an antigen-binding fragment (Fab) that inhibits RAD51–ssDNA binding activity (187). Fab-F2-iPTD binds strongly to RAD51 and enhances cell death in methyl methanesulfonate-treated cells (187). 3E10 and Fab-F2-iPTD are unique in that they are cell permeable, unlike most other antibodies whose size limits them to extracellular targets (188). The ability of 3E10 and Fab-F2-iPTD to penetrate the cell enables improved specificity and binding of RAD51 compared to the small molecules currently in development. Furthermore, tissues with an abundance of extracellular DNA, such as tumors, preferentially uptake 3E10 compared to normal cells (189). However, further study regarding the specificity of many of these antibodies and small molecule inhibitors against cancer cells is still needed.

## CONCLUSIONS AND FUTURE DIRECTIONS

Finding the right balance of RAD51 activity is critical for maintaining genome stability and cancer prevention. Misregulation of RAD51, and its regulators, is associated with FA-like syndrome and cancer, particularly breast and ovarian cancers. The specificity for breast and ovarian cancers is striking and the rationale behind this association remains a mystery. However, there is some speculation that hormone-related cancers may be associated with HR deficiency. A frustrating aspect of studying RAD51 and its regulators in cancer is that variants identified in patients remain VUS; therefore, it is unclear whether or how they contribute to cancer predisposition. Functional analysis and characterization of these VUS will give insight into RAD51’s role in not only cancer, but FA-like syndrome as well. While we know that RAD51 plays a role in FA, the specific link between RAD51 regulation and FA has yet to be fully elucidated. To date, RAD51 and only a subset of its regulators, BRCA2, PALB2, RAD51C and XRCC2, are identified as FA genes. However, given that the RAD51 paralogs interact together in complexes, it is probable that the other RAD51 paralogs may represent heretofore unknown FA-like genes. It is also possible that pharmacologically inhibiting RAD51 could result in FA-like symptoms. Further investigation is needed to determine the precise role of RAD51 and its regulators in FA.

Since RAD51 misregulation contributes to cancer and resistance to therapy, pharmacologically modulating RAD51 activity is an active area of research. It will be important to determine whether modulating a specific RAD51 activity (i.e. protomer interactions, DNA binding, etc.) will be more clinically efficacious and in which tumor types it will benefit most. To date, there is only one active clinical trial investigating the efficacy of directly targeting RAD51. This trial examines the small molecule inhibitor CYT-0851, which has shown promising results in preclinical models (NCT03997968). Here, we focused on RAD51 regulation and examined pharmacological methods used to target RAD51 activity. Developing RAD51 modulators that are safe and effective for clinical use is an exciting approach to target cancer.
